# The association between maternal cytomegalovirus urinary excretion and congenital infection rate

**DOI:** 10.1186/s12884-021-04194-6

**Published:** 2021-11-01

**Authors:** Rita Zlatkin, Efraim Bilavsky, Joseph Pardo, Lina Salman, Ron Bardin, Eran Hadar, Anat Shmueli

**Affiliations:** 1 Sackler Faculty of Medicine, Tel Aviv University, Tel Aviv, Israel; 2 Schneider Children’s Medical Center, Petach Tikva, Israel; 3 Department of Obstetrics and Gynecology, Helen Schneider Hospital for Women, Rabin Medical Center, 49100 Tikva, Israel

**Keywords:** CMV, Cytomegalovirus, Congenital infection, Maternal infection, Urinary excretion

## Abstract

**Background:**

In utero Cytomegalovirus (CMV) vertical transmission occurs predominantly during primary maternal infection. There are no known non-invasive methods for diagnosis of fetal infection before delivery, however some risk factors have been suggested. We aimed to evaluate the association between maternal CMV urinary excretion and congenital CMV infection.

**Methods:**

A retrospective cohort study of all women who were diagnosed with primary CMV infection during pregnancy in a single university affiliated tertiary medical center, between 2012 and 2016. We examined congenital CMV infection and disease rates among infants born to women with and without CMV urinary excretion.

**Results:**

Overall, 126 women were included, 77 in the positive urinary excretion group, and 49 in the negative urinary excretion group. There was no difference in maternal symptoms between the groups. We found no difference in congenital CMV infection and disease rates between infants born to women with and without urinary excretion of CMV (congenital infection rate 37.1% vs. 24.4%, *p* = 0.209, congenital disease rate of 18.2% vs. 22.4%, *p* = 0.648). Women with positive urinary CMV excretion had lower IgG avidity values (36.7% vs 54.6%, *p* = 0.007), with no additional difference in serology pattern. Compared to asymptomatic women, those with CMV related symptoms did not have significantly higher rates of urinary excretion of CMV (70% vs. 60.5%, *p* = 0.38) or congenital infection rates (40.7% vs. 31.2%, *p* = 0.48).

**Conclusion:**

Among infants of women with primary CMV infection in pregnancy, we did not find an association between urinary excretion of CMV and congenital CMV infection.

## Introduction

Cytomegalovirus (CMV) is a DNA herpesvirus, established as the most common congenital viral infection, with birth prevalence of approximately 0.5% [1]. Congenital CMV disease is a leading cause of birth defects, developmental disabilities and sensorineural hearing loss [2].

The most important protective factor against congenital CMV infection is preexisting maternal immunity against the virus [3], as is the timing of infection during gestation: the rate of vertical transmission in women with primary CMV infection increases with advancing gestational age [4–7] - from approximately 40% during first trimester [6] to 73.3% for third trimester maternal infection [7]. Nevertheless, sequelae in the offspring appears to be less severe the later in gestation transmission occurs [8–10]. Other than those, several additional risk factors, associated with vertical transmission to the fetus and severity of congenital CMV disease, have been described for both primary and non-primary maternal infections, such as high-risk occupation for viral exposure, and abnormal fetal MRI [11, 12].

Diagnosis of clinically suspected maternal primary CMV infection is based on seroconversion of negative to positive CMV IgG in maternal serum, alongside with determination of IgG avidity, which is frequently necessary in order to establish the estimated timing of the infection [13, 14]. In order to definitively determine fetal infection, the preferred prenatal analysis is PCR for CMV DNA via amniocentesis [15–17]. In addition, sonographic monitoring for fetal anomalies that may indicate an affected fetus should be conducted, even though these anomalies are not universally diagnostic nor pathognomonic for CMV disease. In the presence of a positive PCR for CMV DNA, these finding are considered indicative for CMV congenital infection. Despite the abovementioned methods, prediction of CMV congenital infection and disease in an infected mother is limited. For that reason, we sought to investigate the role of maternal urinary CMV excretion in these entities.

The medical literature is sparse as to the association between the presence of CMV DNA in maternal urine and the rate of CMV congenital infection and disease. Delforge et al. [18] investigated a similar question and found a marginal statistically significant correlation between the two, however their study addressed only the association between CMV viruria and congenital infection rate, and did not address other implications of maternal CMV urine excretion. Therefore, the aim of our study was to establish whether maternal CMV urinary excretion is associated with congenital CMV infection and disease.

## Materials and methods

### Study population

A retrospective cohort study of all pregnant women who visited the CMV clinic in a single university affiliated tertiary medical center, between 2012 and 2016, and were diagnosed with primary CMV infection during pregnancy. Women were categorized according to CMV urinary excretion: positive urinary excretion group and negative urinary excretion group at time of diagnosis. We excluded women who were diagnosed with non-primary CMV infection, periconceptional CMV infection, multiple gestations and women who participated in a parallel interventional trial for CMV prevention. In addition, women who did not undergo amniocentesis or whose neonatal electronic health record did not contain information on CMV urinary analysis, were also excluded.

### Definitions

Primary maternal CMV infection was defined in women who underwent CMV IgG seroconversion, according to their serology analysis prior to and during pregnancy. If prior IgG status was unknown, IgG avidity was measured to ascertain the estimated timing of the infection [13, 14, 19, 20], and to exclude any suspected peri- or pre-conceptional infections. In some women with IgG seroconversion but otherwise borderline serology patterns, maternal symptoms were also taken into account in determination of primary infection. CMV DNA urine excretion analysis was used to stratify women to the abovementioned study groups.

Congenital CMV infection was defined by positive PCR for CMV DNA in amniocentesis that was performed after 21 gestational weeks and at least 7 weeks after the estimated onset of maternal infection (based on serological conversion and IgG avidity) [21], or by detection of CMV in neonatal urine analysis, which was performed immediately after birth. Congenital CMV disease was defined for any fetus with sonographic or MRI findings that are considered suggestive of a congenital disease, as well as for any neonate with signs of sensorineural hearing loss, as evaluated by a post-partum brainstem Evoked Response Audiometry (BERA), preformed shortly after birth.

Maternal acute CMV infection symptoms were collected as well, including mononucleosis-like symptoms such as fever and malaise, and elevated liver enzymes [22].

### Data collection

Data was retrieved from the computerized comprehensive database of our CMV Clinic. The following demographic and CMV related maternal properties were recorded: age, gravidity, parity, any maternal chronic diseases, CMV serology status during and prior to conception (including IgG, IgM, IgM VIDAS and IgG Avidity), urinary qualitative PCR for CMV detection, maternal symptoms (fever, malaise) and laboratory results (Aspartate Transaminase [AST], Alanine Transaminase [AST]) during acute CMV infection, gestational age at CMV infection diagnosis, as well as gestational age at urine analysis and amniocentesis.

Maternal urinary tests for CMV were performed during the first visit in our CMV clinic, which was scheduled as soon as maternal primary infection was diagnosed by serology for 6 weeks after diagnosis at the latest. Only one test was taken from each woman, in order to avoid different time points that may have caused a bias in test interpretation.

The following obstetrical, fetal and neonatal parameters were recorded: gestational age at delivery, mode of delivery, birth weight, gender, PCR results for CMV DNA in amniocentesis, neonatal urine analysis via PCR for CMV detection and neonatal BERA test.

Imaging studies are routinely performed among primary CMV infected women in order to assess the probability of symptomatic congenital disease of the fetus and newborn. We collected sonographic and brain MRI fetal findings which are typical (but not pathognomonic or specific) for congenital CMV disease, including bilateral periventricular calcifications (hyperechoic foci), intraventricular adhesions, cerebral ventriculomegaly, microcephaly, fetal growth restriction, hyperechogenic fetal bowel, hepatic calcifications and hepatosplenomegaly [21, 23–27].

### Outcome measures

The primary outcome measures of the study were defined as congenital CMV infection in the positive versus negative urinary excretion of CMV, determined by either CMV-positive amniocentesis or neonatal urine and as congenital CMV disease, determined by typical sonographic or MRI findings and/or by neonatal abnormal BERA test results.

### Statistical analysis

Statistical analysis was generated using SAS Software, Version 9.4. Continuous variables were presented by mean and standard deviation. Categorical variables were presented by number and percentage. Categorical data were analyzed using Fisher’s exact test and continuous variables were compared using Mann–Whitney–Wilcoxon test as appropriate. Two-sided *p* values less than .05 were considered statistically significant.

### Ethics

The study was approved by the local Institutional Review Board at Rabin Medical Center, approval no. RMC-13-0237.

## Results

Overall, during the study period, 1632 women visited the CMV clinic. One hundred and eighty-two (11.15%) women were diagnosed with primary CMV infection. Of them, 56 were excluded: 22 did not have maternal urinary CMV analysis results, 18 had periconceptional CMV infection, 7 participated in another clinical trial, 8 had no neonatal CMV urinary analysis or amniocentesis results and one had a multiple gestation. Eventually, 126 women met the inclusion criteria and were divided into two study groups according to CMV urinary excretion: 77 (61.11%) women with positive urinary excretion and 49 (38.89%) with negative urinary excretion. Demographic and baseline maternal characteristics are presented in Table 1. Women with positive urinary CMV excretion were slightly, although significantly, older than the non-excreting group (31.2 vs. 29.7 years, *p* = 0.043), with no other significant differences in gravidity, parity and maternal chronic diseases.

**Table 1:** Baseline characteristics of the study population

Parameter	Positive Urinary CMV (*n* = 77)	Negative Urinary CMV (*n* = 49)	*p*-value
Age, years	31.2 ± 3.4	29.7 ± 4.4	0.043
Gravidity	2.5 ± 1	2.4 ± 1.3	0.901
Parity	1.2 ± 0.8	1.2 ± 1	0.824
Nulliparity	9 (11.7%)	8 (16.3%)	0.593
Maternal chronic disease^a^	13 (16.9%)	9 (18.4%)	0.42

Data presented as mean ± standard deviation for continuous variables and as n(%) for categorical variables

^a^ includes asthma, hypothyroidism, epilepsy, chronic hypertension and diabetes mellitus

Among infants diagnosed with in utero CMV infection (overall 36 cases), 23 were diagnosed based on positive CMV PCR, and 24 had CMV urinary excretion detected after birth in the newborn infant. 11 cases had both CMV PCR and CMV urinary excretion. We found that the congenital CMV infection rate was higher in the positive urinary excretion group (33.8% vs. 20.4% In the non-excretion group, *p* = 0.209), however the congenital CMV disease rate was lower in this group (18.2% vs. 22.4%, *p* = 0.648). These findings were not statistically significant.

Maternal CMV serology patterns are presented in Table 2. We found a significant difference in CMV Avidity between the two groups - the positive urinary CMV excretion had higher rates of low avidity compared to the non-excretion group (54.6% vs. 36.7%, *p* = 0.007). Non-significant trends demonstrated similar IgG (81.8% vs. 87.8% in the non-excretion group, *p* = 0.458) and IgM positive VIDAS (an automated enzyme-linked fluorescent immunoassay, for the detection CMV IgM antibodies) (78% vs. 85.8% in the non-excretion group, *p* = 0.399) rates, as well as similar gestational age at CMV infection diagnosis (17.4 vs. 16.4 weeks in the positive excretion group, *p* = 0.464).

**Table 2:** CMV Infection related maternal properties

Parameter		Positive Urinary CMV (*n* = 77)	Negative Urinary CMV (*n* = 49)	*p*-value
CMV IgG		63 (81.8%)	43 (87.8%)	0.458
CMV IgM		74 (96%)	46 (94%)	0.677
CMV IgM VIDAS^1^		60 (78%)	42 (85.8%)	0.399
CMV Avidity^1^	Low	42 (54.6%)	18 (36.7%)	0.007
	Moderate/High	17 (22%)	24 (49%)	
	Index	0.3 ± 0.16	0.36 ± 0.22	0.229
	Missing Data	18 (23.4%)	7 (14.3%)	
Maternal Symptoms	Overall	21 (27.3%)	9 (18.4%)	0.388
	Fever	14 (19.4%)	8 (20%)	1.00
	Malaise	12 (16.7%)	8 (20%)	0.797
	Elevated Liver Enzymes	2 (2.8%)	3 (7%)	0.358
Gestational age at CMV diagnosis, weeks		17.4 ± 6.3	16.4 ± 6.6	0.464
Gestational age at maternal urine test, weeks		22.8 ± 6.7	23.3 ± 6.2	0.381

Data presented as mean ± standard deviation for continuous variables and as n(%) for categorical variables

^1^Data was missing in some of the women in the study groups

We also compared separately sonographic and MRI findings that are typical for congenital CMV infection between the two study groups (Table 3). Positive MRI findings rates (8.1% vs. 5.2% in the positive excretion group, *p* = 0.391), and sonographic typical findings (10.2% vs. 7.8% in the positive excretion group, *p* = 0.746) were comparable between the groups.

**Table 3:** Pregnancy and neonatal outcome

Parameter		Positive Urinary CMV (*n* = 77)	Negative Urinary CMV (*n* = 49)	*p*-value
Gestational age at delivery, weeks		38.6 ± 3.7	38.9 ± 1.7	0.588
Mode of delivery^a^	NVD	53 (68.9%)	36 (73.5%)	0.202
	CD	12 (15.6%)	3 (6.1%)	
	OVD	3 (3.9%)	4 (8.2%)	
	Missing Data	2 (2.6%)	3 (6.1%)	
Termination of Pregnancy		7 (9%)	3 (6.1%)	0.202
Birth weight, grams		3237.9 ± 382.8	3259.5 ± 437.4	0.792
Neonatal gender, male		34 (44.2%)	21 (42.9%)	0.842
Congenital CMV infection^a, b^	Positive	26 (33.8%)	10 (20.4%)	0.209
	Missing Data	7 (9%)	3 (6.1%)	
Sonographic fetal findings^a^	Positive	6 (7.8%)	5 (10.2%)	0.746
	Missing Data	8 (10.4%)	7 (14.3%)	
MRI findings^a^	Positive	4 (5.2%)	4 (8.1%)	0.391
	Missing Data	7 (9%)	3 (6.1%)	
Congenital CMV Disease^c^		14 (18.2%)	11 (22.4%)	0.648

Data presented as mean ± standard deviation for continuous variables and as n(%) for categorical variables

*NVD* Normal Vaginal Delivery; *CD* Cesarean Delivery; *OVD* Operative Vaginal Delivery; *TOP* Termination of Pregnancy

^a^Data was missing in some of the women in the study groups

^b^Determined by amniocentesis or neonatal urine test

^c^Defined by clinical neonatal findings and typical sonographic and fetal MRI findings

An additional analysis was made to examine a possible difference in CMV excretion patterns and congenital CMV infection rate between symptomatic and asymptomatic women. Symptomatic women had a non-significant trend of higher rates of both maternal urinary excretion (70% vs. 60.5%, *p* = 0.388) and congenital infection rates (40.7% vs. 31.2%, *p* = 0.400). We investigated different obstetric parameters and found that both groups had similar rates of termination of pregnancy, gestational age at delivery and neonatal birth weight.

## Discussion

In the current study we aimed to investigate the association between maternal urinary CMV excretion and the rate of congenital CMV infection and disease, among women with primary CMV infection. Our findings reveal no significant association between maternal urinary CMV excretion and fetal congenital infection or disease, thereupon weakening the assumption that it might have a possible role as a predictive factor for congenital infection. Nevertheless, our study raises some interesting issues which are discussed below, and warrant further evaluations in large scale studies. CMV is shed in the urine and other bodily fluids for months after seroconversion [28], or even years in cases of infants with a diagnosis of congenital infection. The rate of CMV urinary excretion differs according to the week of infection and the week of urine analysis [29], and therefore varies between different studies. Some studies reported a rate of 3–5% [30, 31] while others reported CMV viruria as high as 13% [29, 32]. In comparison, our study demonstrated that the rate of positive CMV excreting women was significantly higher (61.11%), possibly due to our inclusion of women with exclusively primary infection. We should also be mindful to the fact that maternal CMV urinary shedding increases with gestation [28], and diagnosis of CMV infection occurred a week later among the women with positive urinary excretion (17.4 weeks of gestation vs. 16.4 in women without CMV urinary excretion). Regardless, it is possible that our results imply that CMV urinary shedding is more prevalent in women with primary infection than in those with re-infection or re-activation, however the latter were excluded from our study.

Efforts are made in order to better predict congenital CMV infection and disease in infants born to women who were infected with the virus, especially during the first trimester of pregnancy. The role of CMV urinary excretion is not well studied as a possible predictor for these outcomes and is mainly considered as non-related to intrauterine vertical transmission of the virus. Lazzarotto et al. [33], in a comprehensive review of new advances in congenital CMV infection diagnosis, described the secondary role of virological testing of maternal secretions and reported low positive prediction rates of 57.1% for congenital CMV infection and disease in infants of women who excreted CMV in the urine in the first two trimesters of pregnancy. However, in this study there was no stratification according to primary and non-primary infection.

Our results show that women with CMV viruria had a significantly higher rate of low avidity in comparison to women without presence of CMV in their urine. These results raise the possibility that urinary CMV excretion is associated with a more recent infection. Our results point to the possibility that infants of women who shed CMV in their urine were probably infected more recently and might have a higher risk for congenital CMV infection than women who do not excrete the virus in their urine.

After diagnosis of a congenital CMV infection is made, further evaluation is required in order to predict the likelihood and severity of congenital disease. This includes imaging studies, mainly sonographic but recently also with fetal MRI [34, 35], which all aim to better predict possible neurodevelopmental delays by identifying typical findings [21, 23–25, 36].. As part of our intent to evaluate the association between maternal urinary CMV excretion and congenital disease, we investigated possible differences in imaging findings between the two study groups, under the assumption that if urinary CMV excretion increases the risk for fetal infection there might also be more sonographic and MRI abnormalities in these fetuses. Our results show no significant differences between the groups, neither when comparing the sonographic and MRI findings nor when comparing neonatal sensorineural hearing loss, therefore denoting that we could not establish an association.

Less than 5% of women infected with CMV are symptomatic [37]; however, the symptoms - such as malaise, fever and fatigue - are highly non-specific for CMV infection. Having said that, we should bear in mind that it is possible that symptoms that were reported by the women in our study were actually non-CMV related.

The strength of our study lies in the specific selection of a maternal population with primary CMV infection, and the selection of a scarcely investigated association in current literature. Additionally, the study was conducted in a single large tertiary medical center, with a high quality dedicated CMV clinic that was the key coordinator of follow up and evaluation of the patients who participated in the study. The main limitation of the study is the relatively small sample size. Due to our exclusion criteria and the sometimes-hard diagnosis of accurate timing of infection, we had to exclude a relatively large number of women. Importantly, urinary CMV shedding may be intermittent and/or prolonged [38] - therefore causing bias.

In conclusion, among women with primary CMV infection in pregnancy, we could not find an association of either urinary excretion of CMV and symptomatic maternal CMV infection, with a higher risk for congenital CMV infection and disease. However, our findings do reveal that urinary CMV excretion is associated with a more recent infection. Further studies with larger study groups are needed in order to establish these conclusions.

## Data Availability

The datasets used and/or analysed during the current study are available from the corresponding author on reasonable request.
